# Exploring the universe through dusty visions

**DOI:** 10.1098/rsta.2023.0210

**Published:** 2024-06-23

**Authors:** Nozair Khawaja, Fabian Klenner, Jamey Szalay, Masanori Kobayashi, Christelle Briois, Ingrid Mann

**Affiliations:** ^1^ Department of Planetary Sciences and Remote Sensing, Freie Universität Berlin, Berlin 12249, Germany; ^2^ Institute of Space Systems, University of Stuttgart, Stuttgart 70569, Germany; ^3^ Department of Earth and Space Sciences, University of Washington, Seattle, WA 98195, USA; ^4^ Department of Astrophysical Sciences, Princeton University, Princeton, NJ 08544, USA; ^5^ Planetary Exploration Research Center, Chiba Institute of Technology, Chiba 275-0016, Japan; ^6^ Laboratory of Physics and Chemistry of the Environment and Space, UMR-CNRS-University of Orléans, Orléans 45071, France; ^7^ Department of Physics and Technology, UiT Norwegian Arctic University, 9037, Norway

**Keywords:** cosmic dust, solar system, interstellar medium, meteorites, asteroids and comets, icy moons

Dust in space is a universal phenomenon that can be observed within our cosmic neighbourhood. Examples include the dust from the surface of the moon to the further reaches of the outer solar system, such as Saturn’s rings, and even beyond in the galactic environment. Dust in the universe has constantly challenged astronomers’ views of heavenly bodies and phenomena, often obscuring light coming from those objects. With the advent of science and technology, followed by the development of modern instruments, cosmic dust is now considered an important source of information that helps to decipher the composition, evolution and formation histories of distant bodies across the universe. The nature of physico-chemical phenomena of unreachable objects and locations in the universe can be investigated by sampling dust in the solar system, much like photons of light captured from distant galaxies [1]. Typically, cosmic dust consists of particles ranging in size from nano-metres to millimetres. This dusty material is incorporated into comets, asteroids and meteorites during the evolution of the protoplanetary systems, and continually evolves on the surfaces of all airless bodies.

Dust can be distinguished in terms of its composition, size and dynamical properties, which are indicative of the grains’ origins: comets, asteroids, airless moons and interstellar medium. The extinction, or scattering, of light from dust clusters in the interstellar medium (ISM) was first realized in 1930 (cf. [2]). Since then, the reddening of distant stars has been observed through temporary decreases in stellar flux, a process known as interstellar reddening. This phenomenon occurs when dust particles absorb photons in the line of sight and re-emit light at a longer wavelength. Heavier elements, created through nuclear fusion in stellar cores, feed dust particles in the ISM. This is particularly notable given the fact that elements heavier than helium are thought to reside in ISM dust grains (cf*.* [3]). Infrared radiation in ISM is absorbed particularly strongly by dust grains, resulting in absorption lines in the light that reaches Earth [4]. These absorption lines can be measured with spectroscopic methods and provide some compositional information about dust grains. Moreover, cosmic dust holds secrets about the early stages of the universe, including information about organic molecules that may have played a role in the origin of life on Earth. Approximately 100–300 tonnes [5] of cosmic dust enter Earth’s atmosphere daily, leading to the creation of not only extensive scientific collections of micrometeorites (MMs) but also collections for wider society held at museums.

In recent decades, (inter)planetary space missions have carried a number of instruments, so-called dust telescopes (or dust instruments), to examine different dust environments within the solar system. Helios, Galileo, Ulysses and Cassini–Huygens were interplanetary space missions, which detected interplanetry and interstellar dust through onboard dust instruments. Over 30 years ago, the Giotto spacecraft had a close encounter with comet Halley and observed dust and volatile emissions, with organic compounds being particularly abundant [6,7]. During the mission life time, Ulysses revealed a higher abundance of dust particles entering the solar system and also recognised different populations of interplanetary dust particles with the onboard dust experiment [8]. Until 2017, Cassini observed, collected and analysed dust particles during its traversals of Saturn’s rings and flybys of the planet’s moons. Cassini’s Cosmic Dust Analyzer (CDA; [9]), a dust mass spectrometer, detected 36 interstellar dust particles as they passed through the Saturnian system [10]. These homogenized silicate grains with iron inclusions were depleted of carbon-bearing compounds, indicating their processing in the ISM. The same instrument constrained Saturn’s moon Enceladus to be a likely habitable place through the analysis of icy dust grains that are actively emitted from the moon [11,12]. Later, in 2016, the Rosetta spacecraft made several flybys of 67P/Churyumov–Gerasimenko and observed outbursts of dust and volatiles. The onboard dust observatories Cosmetary Secondary Ion Mass Analyzer [13] and Rosetta Spectrometer for Ion and Neutral Analysis [14] were able to collect volatile species alongside micron- to millimetre-sized icy dust particles. From 2013–2014, NASA’s LADEE (Lunar Atmosphere and Dust Environment Explorer) mission orbited the moon and made comprehensive in-situ measurments of the moon’s impact ejecta dust environment with the Lunar Dust Experiment [15]. More recently, sample return missions from two near-Earth asteroids were completed by JAXA and NASA. While Hayabusa2 visited 162 173 Ryugu, OSIRIS-REx brought back samples from 101 955 Bennu. Based on the results from JAXA’s and NASA’s sample return missions, the Stardust mission, LADEE, laboratory experiments and modelling efforts have revealed further insights into cosmic dust. Upcoming observations from DESTINY+ [16] and the Interstellar Mapping and Acceleration Probe [17] will continue the legacy of in-situ dust analysis with advanced in-situ dust composition analysis.

Review articles and collections on cosmic dust were compiled as early as 1978, when the study of cosmic dust was still a relatively new field, focusing mainly on interstellar dust, comets, meteors, lunar dust, zodiacal light, dust dynamics, and laboratory simulations. Later collections at the turn of the 21st Century highlighted the growth of the field over the previous decades: near-Earth dust, dusty rings around planets, and also dynamical models of interplanetary dust [1]. Indeed, contemporary cosmic dust research spans a wide range of subtopics, far too broad to provide a complete overview in this collection. Therefore, the scope of this special issue focuses only on a small subset of topics and is not intended to represent cosmic dust studies as a whole. The articles in this collection *Dust in the Solar System and Beyond*, published in *Philosophical Transactions of the Royal Society A* are dedicated to some of the latest results described below.

The first two papers focus on the detection and analysis of meteors and meteorites. The opening paper by Feige *et al.* [18] investigates the precise origins of 12 MMs to constrain whether MMs found in urban areas or Antarctica originated from comets or asteroids. This study measures concentrations of aluminium-26 and beryllium-10 via mass spectrometry and compares the results with a model concerning the transport and irradiation of precursors of these meteorites in space. The study successfully differentiates MMs into two populations, with one type originating from the inner solar system and the other belonging to its outer reaches. The second follow-up paper by van Ginneken *et al.* [19] is a review describing the current status of the collections of MMs on Earth. The paper describes a number of collection sites of MMs, including Antarctica and the deep sea alongside the Atacama Desert. This review provides an overview about these collected MMs and their use by scientific communities to address specific research questions. The authors discuss the status of research towards finding large fossil MMs and also advancements in the collection of airborne cosmic dust.

The next four papers in our collection refer to the *in situ* detection of dust particles in interplanetary space. The third paper by Simolka *et al.* [20] presents a space-based dust analyser (mass spectrometer), the DESTINY+ Dust Analyzer, that will fly aboard JAXA’s DESTINY+ (Demonstration and Experiment of Space Technology for INterplanetary voYage with Phaethon fLyby and dUst Science) interplanetary space mission. This paper provides technical details of the instrument and demonstrates its effectiveness in detecting and analysing the composition of dust during the flyby of the asteroid 3200 Phaethon. The paper describes the functional principle of the instrument, which uses impact ionization akin to its heritage, CDA that flew on board the Cassini spacecraft. In the fourth paper by Krüger *et al*. [21], the authors use an Interplanetary Meteoroid Environment for eXploration dust stream model and predict cometary stream particles along the orbit of the Ulysses spacecraft that was launched in 1990 and also carried an impact ionization dust detector. The study reports that the detection of 19 particles in the micrometre size range by Ulysses originated from five different comets. The traceback of dust particles to their origin provides a new opportunity to understand *in situ* compositional analysis of these grains by future space missions, such as JAXA’s DESTINY+. The fifth perspective review paper by Sommer M. [22] describes the detection of dust clusters in near-Earth space within the terrestrial magnetosphere by an impact ionization detector onboard the HEOS-2 satellite. The author highlights the electrostatic interaction between dust clusters and Earth’s magnetosphere and suggests that this phenomenon can be used as a novel method to measure dust in near-Earth orbit. The paper emphasizes a measurement campaign to investigate the origin of these dust swarms by future space missions with dust detectors such as JAXA/DLR DESTINY+ mission. The sixth paper by Kearsley *et al.* [23] discusses the hypervelocity impact of cosmic dust particles onto the Hubble Space Telescope (HST) in low-Earth orbit. In contrast to the previous three papers, the research here focuses on larger particles (millimetre to centimetre scale) incident upon the radiator shield of HST. Via the complementary use of laboratory experiments, the authors analysed images of impactors roughly 50 μm in size and found that their composition is similar to ordinary chondrite meteorites. The study also compares results with earlier models, Orbital Debris Engineering Model and Meteoroid and Space Debris Terrestrial Environment Reference developed by NASA and ESA, respectively, and discusses the mismatch of their results with the presented study.

The last two papers concern laboratory simulations that aim to address questions related to icy dust grains ejected from subsurface ocean-bearing icy moons in the solar system. The seventh paper by Spesyvyi *et al*. [24] describes the development of an accelerator to simulate the flow of submicron-sized water ice particles, named the SElected Ice Nanoparticle Accelerator (SELINA). This work is critical for spaceborne impact ionization mass spectrometers that explore the habitability of subsurface oceans on icy moons in the outer solar system. In this study, the kinetic energy per charge was set up to 200 eV to achieve velocities of icy particles up to 600 m s^−1^. Thus far, the instrument has accelerated positively and negatively charged ice particles containing aqueous solutions of sodium chloride below 0.2 M. The study also demonstrates the capability of SELINA to record time-of-flight mass spectra of 120 nm particles accelerated up to hypervelocities of 3000 m s^−1^. The final paper in this collection by Khawaja *et al*. [25] is dedicated to the detection of potential biosignature compounds in the icy dust particles emerging from the subsurface ocean of Saturn’s moon Enceladus. In this work, triglycine peptide was hydrothermally processed at approximately 80 bar and 80°C to simulate conditions at the ocean-core boundary of Enceladus. With this newly established experiment, the authors demonstrate the effect of the hydrothermal processing on the synthesis of new species and also the degradation of triglycine itself. The samples of these simulations were measured with Laser Induced Liquid Beam Ion Desorption mass spectrometry, which is a laboratory analogue for impact ionization mass spectrometry of ice grains in space employed by instruments like Cassini’s CDA [9] and Europa-Clipper’s SUrface Dust Analyzer [26] .

## Data Availability

This article has no additional data.
